# Supplementary material for: From sequence to enzyme mechanism using multi-label machine learning
Source: BMC Bioinformatics. 2014 May 19;15:150. doi: 10.1186/1471-2105-15-150 (PMC4229970; doi:10.1186/1471-2105-15-150)
Supplement: Additional file 2 — Java code of ml2db. Additional file ml2db_code.tar.gz contains the Java source code to run the multi-label machine learning experiments and save the results to database. The code’s Javadoc is included. [file 1471-2105-15-150-S2.zip › additional file 2/ml2db/ml2db/doc/mulan/dimensionalityReduction/BinaryRelevanceAttributeEvaluator.html]

BinaryRelevanceAttributeEvaluator


JavaScript is disabled on your browser.


- Overview
- Package
- Class
- Use
- Tree
- Deprecated
- Index
- Help

- Prev Class
- Next Class

- Frames
- No Frames

- All Classes

- Summary:
- Nested |
- Field |
- Constr |
- Method

- Detail:
- Field |
- Constr |
- Method


mulan.dimensionalityReduction

## Class BinaryRelevanceAttributeEvaluator

- java.lang.Object
- - weka.attributeSelection.ASEvaluation
  - - mulan.dimensionalityReduction.BinaryRelevanceAttributeEvaluator

- All Implemented Interfaces:
  :   java.io.Serializable, weka.attributeSelection.AttributeEvaluator, weka.core.CapabilitiesHandler, weka.core.RevisionHandler

  ---

    

  ```
  public class BinaryRelevanceAttributeEvaluator
  extends weka.attributeSelection.ASEvaluation
  implements weka.attributeSelection.AttributeEvaluator
  ```

  Author:
  :   George Traianos, Grigorios Tsoumakas

  See Also:
  :   Serialized Form

- - ### Nested Class Summary

    Nested Classes

    | Modifier and Type | Class and Description |
    | --- | --- |
    | `class` | `BinaryRelevanceAttributeEvaluator.Rank` a wrapper class for score-based attribute ranking |
  - ### Constructor Summary

    Constructors

    Constructor and Description

    `BinaryRelevanceAttributeEvaluator(weka.attributeSelection.ASEvaluation ase,
    MultiLabelInstances mlData,
    java.lang.String combapp,
    java.lang.String norm,
    java.lang.String mode)`
  - ### Method Summary

    Methods

    | Modifier and Type | Method and Description |
    | --- | --- |
    | `double` | `average(double[][] scoreList, int index)` average score combination approach |
    | `void` | `buildEvaluator(weka.core.Instances data)` Not supported |
    | `static double[]` | `dl(double[] array)` divide by length (dl) normalization |
    | `static double[]` | `dm(double[] array)` divide by maximum (dm) normalization |
    | `double` | `evaluateAttribute(int attribute)` Evaluates an attribute |
    | `double` | `highest(double[][] scoreList, int index)` highest score combination approach |
    | `double` | `lowest(double[][] scoreList, int index)` lowest score combination approach |
    | `static double` | `norm(double[] vector)` calculates the norm of a vector |
    | `static void` | `normalize(double[] array)` normalizes an array (in the range of [0,1]) |
    | `int[]` | `order(int[] ranking)` orders the ranking scores according to their attributes' original indices |
    | `int[]` | `rankAsc(double[] scores)` returns a ranking of attributes (where each attribute is represented by its index) |
    | `int[]` | `rankDesc(double[] scores)` returns a ranking of attributes (where each attribute is represented by its index) |

    - ### Methods inherited from class weka.attributeSelection.ASEvaluation

      `forName, getCapabilities, getRevision, makeCopies, postProcess, runEvaluator`
    - ### Methods inherited from class java.lang.Object

      `equals, getClass, hashCode, notify, notifyAll, toString, wait, wait, wait`

- - ### Constructor Detail


    - #### BinaryRelevanceAttributeEvaluator

      ```
      public BinaryRelevanceAttributeEvaluator(weka.attributeSelection.ASEvaluation ase,
                                       MultiLabelInstances mlData,
                                       java.lang.String combapp,
                                       java.lang.String norm,
                                       java.lang.String mode)
      ```

      Parameters:
      :   `ase` -
      :   `mlData` -
      :   `combapp` - combination approach mode ("max", "avg", "min")
      :   `norm` - normalization mode ("dl", "dm", "none")
      :   `mode` - scoring mode ("eval", "rank")
  - ### Method Detail


    - #### rankAsc

      ```
      public int[] rankAsc(double[] scores)
      ```

      returns a ranking of attributes (where each attribute is represented by its index)

      Parameters:
      :   `scores` - the attributes' scorelist

      Returns:
      :   an ascending ranking of the attributes, based on their scores


    - #### rankDesc

      ```
      public int[] rankDesc(double[] scores)
      ```

      returns a ranking of attributes (where each attribute is represented by its index)

      Parameters:
      :   `scores` - the attributes' scorelist

      Returns:
      :   a descending ranking of the attributes, based on their scores


    - #### order

      ```
      public int[] order(int[] ranking)
      ```

      orders the ranking scores according to their attributes' original indices

      Parameters:
      :   `ranking` - a rank table

      Returns:
      :   the order of the ranking scores


    - #### highest

      ```
      public double highest(double[][] scoreList,
                   int index)
      ```

      highest score combination approach

      Parameters:
      :   `scoreList` - all attributes' score lists
      :   `index` - the index of a specific attribute's score

      Returns:
      :   the highest score achieved in any of the the input score lists


    - #### lowest

      ```
      public double lowest(double[][] scoreList,
                  int index)
      ```

      lowest score combination approach

      Parameters:
      :   `scoreList` - all attributes' score lists
      :   `index` - the index of a specific attribute's score

      Returns:
      :   the lowest score achieved in all of the input score lists


    - #### average

      ```
      public double average(double[][] scoreList,
                   int index)
      ```

      average score combination approach

      Parameters:
      :   `scoreList` - all attributes' score lists
      :   `index` - the index of a specific attribute's score

      Returns:
      :   the average score achieved in all the score lists


    - #### norm

      ```
      public static double norm(double[] vector)
      ```

      calculates the norm of a vector

      Parameters:
      :   `vector` - a numeric array (as a vector)

      Returns:
      :   the norm of the given vector


    - #### normalize

      ```
      public static void normalize(double[] array)
      ```

      normalizes an array (in the range of [0,1])

      Parameters:
      :   `array` - a numeric array


    - #### dl

      ```
      public static double[] dl(double[] array)
      ```

      divide by length (dl) normalization

      Parameters:
      :   `array` - a numeric array

      Returns:
      :   a dl normalized copy of array


    - #### dm

      ```
      public static double[] dm(double[] array)
      ```

      divide by maximum (dm) normalization

      Parameters:
      :   `array` - a numeric array

      Returns:
      :   a dm normalized copy of array


    - #### evaluateAttribute

      ```
      public double evaluateAttribute(int attribute)
                               throws java.lang.Exception
      ```

      Evaluates an attribute

      **Specified by:**
      :   `evaluateAttribute` in interface `weka.attributeSelection.AttributeEvaluator`

      Parameters:
      :   `attribute` - the attribute index

      Returns:
      :   the evaluation

      Throws:
      :   `java.lang.Exception`


    - #### buildEvaluator

      ```
      public void buildEvaluator(weka.core.Instances data)
                          throws java.lang.Exception
      ```

      Not supported

      **Specified by:**
      :   `buildEvaluator` in class `weka.attributeSelection.ASEvaluation`

      Parameters:
      :   `data` -

      Throws:
      :   `java.lang.Exception`


- Overview
- Package
- Class
- Use
- Tree
- Deprecated
- Index
- Help

- Prev Class
- Next Class

- Frames
- No Frames

- All Classes

- Summary:
- Nested |
- Field |
- Constr |
- Method

- Detail:
- Field |
- Constr |
- Method
